# Factors Influencing Public Panic During the COVID-19 Pandemic

**DOI:** 10.3389/fpsyg.2021.576301

**Published:** 2021-02-12

**Authors:** Xiangtian Nie, Kai Feng, Shengnan Wang, Yongxin Li

**Affiliations:** ^1^North China University of Water Resources and Electric Power, Zhengzhou, China; ^2^Institute of Psychology and Behaviour, Henan University, Kaifeng, China

**Keywords:** COVID-19, panic, pandemic-related knowledge, self-efficacy, risk, objective social support

## Abstract

The coronavirus disease 2019 (COVID-19) pandemic has been regarded as a public health emergency that caused a considerable degree of public panic (such as anxiety and insomnia) during its early stage. Some irrational behaviors (such as excessive search for information related to the pandemic and excessive hoarding of supplies) were also triggered as a result of such panic. Although there has been plenty of news coverage on public panic due to the outbreak, research on this phenomenon has been limited. Since panic is the main psychological reaction in the early stage of the pandemic, which largely determines the level of psychological adaptation, time of psychological recovery, and the incidence of PTSD, there exists a demand to conduct investigation on it. From a public governance perspective, the government’s assessment of public panic may affect the efficiency and effectiveness of pandemic prevention and control. Therefore, it is of obvious practical significance to investigate public panic during the COVID-19 pandemic and analyze its influential factors. The self-compiled COVID-19 Social Mentality Questionnaire was used to collect data from a total of 16,616 participants online, and 13,511 valid responses were received. The results from the chi-square test showed that there were differences in gender, educational level, age, pandemic-related knowledge, self-efficacy, risk level, and objective social support. Furthermore, multiple linear regression analysis results showed that self-efficacy, gender, educational level, age, risk level, pandemic-related knowledge, and objective social support were significant predictors of public panic. Among the research variables, self-efficacy, gender, educational level, and age were negative predictors of panic while risk level, pandemic-related knowledge, and objective social support were positive predictors of panic.

## Introduction

The coronavirus disease 2019 (COVID-19) is a respiratory infection transmitted by airborne droplets and close contact caused by severe acute respiratory syndrome coronavirus 2 (SARS-CoV-2). The World Health Organization (WHO) declared the novel coronavirus pneumonia outbreak a “public health emergency of international concern” on January 31, 2020, Beijing time. As of May 24, there were 5.29 million confirmed cases and 342,306 deaths worldwide. COVID-19 not only threatens people’s health and safety, but also has a profound impact on people’s lives and work.

The COVID-19 pandemic typically brings stress to the general public. According to the stress theory, stress refers to a series of physiological, psychological and behavioral reactions that occur when people face harmful substances, threats, and challenges inside and outside, and know that such stimuli will pose a threat to them after their own subjective evaluation (Folkman and Lazarus, 1984). The causes of these reactions are called stressors. Studies have pointed out that in various major stress events, infectious diseases not only have a more lasting impact on human physiology, but also produce a variety of adverse psychological reactions in victims (Li and Hua, 2003). In the face of the pandemic, people often experience negative emotions such as anxiety, fear, depression and, in severe cases, some somatic symptoms (Chinese Association for Mental Health, 2020), which have negative impacts on people’s physical and mental health as well as their future life. Although there has been a lot of news about panic during the pandemic, there have been very few studies on public panic. Since panic is an important psychological response to COVID-19, it largely determines the level of subsequent psychological adaptation, time of psychological recovery, and incidence of PTSD. Therefore, in order to overcome the novel COVID-19 as soon as possible, it requires not only the efforts of medical researchers and the professional treatment of front-line medical staff, but also the trusted scientific evidence and knowledge of psychological researchers to provide the public with science-based guidelines to establish a positive social mentality to avoid excessive panic caused by the pandemic (Castelnuovo et al., 2020).

The COVID-19 pandemic, as a public health emergency, is also an emergent risk event for the public. It has the typical characteristics of high-risk events and causes a considerable degree of panic in the society (such as anxiety and insomnia etc.). According to the risk perception theory (Slovic, 1987), a risk event can be interpreted as a “signal”; the nature of the “signal” and the conditions of its transmission process will influence the receivers’ feelings and reactions toward the event. Usually, people rely on their intuition to know and make judgments about risk events, which is called risk perception. Svenson (1988) proposed the mental model of risk, believing that individuals will form different mental models due to their differences in knowledge, experience and other individual characteristics, thus forming a unique perception and value judgment of risk events. Therefore, in the process of forming public risk perception from a risk signal, two factors are involved. First, the characteristics of risk events themselves; second, the receivers’ personal characteristics, such as their personality or cognitive biases. The interaction among these characteristics will also produce a certain effect. People’s risk perception of crisis events can be measured from two dimensions: familiarity and controllability. The high risk end is perceived as “unknown and uncontrollable.” Previous studies found that if risk factors can be classified according to their nature, their risk characteristic dimensions can be significantly correlated. For example, a factor perceived as a voluntary risk is often perceived as controllable; a risk factor perceived as unknown is often perceived as a factor of high anxiety. In the case of COVID-19, on the one hand, the massive, collective stressor of a pandemic far exceeds the capacity of individuals and communities to respond; on the other hand, as COVID-19 is an emerging infectious disease, information about its source, post-infection detoxification time, and pathogenesis is still unclear, which further exacerbates its “uncontrollability.” In previous studies on SARS, Shi and Hu (2004) pointed out that information related to the pandemic, such as etiology, route of transmission and cure rate, is an effective indicator to reflect the controllability of the pandemic, which can significantly affect people’s risk perception. Xie et al. (2005) pointed out that the individual characteristics of the public, such as gender, educational background, personality and relevant knowledge, are the conditions for the “signal” transmission process of risk events. They affect the individual’s ability and willingness to accept risk events, and when such exceeds the individual’s tolerance, it will produce adverse psychological reactions such as panic and even lead to some irrational behaviors, such as excessive search for information related to the pandemic, excessive hoarding of food, and blind use of drugs. Therefore, this study intends to explore the influence of factors such as gender, educational background, risk level, social support, pandemic knowledge and self-efficacy on public panic during the pandemic period. This study aims to analyze the causes of public panic by exploring the influencing factors, help the government in conducting counseling and achieving control, and lay a foundation for subsequent psychological reconstruction.

## Materials and Methods

### Samples

In this study, a convenient sampling method was adopted to carry out a survey in Henan province using the online platform wjx.cn from 17:00 January 27, 2020 to 17:00 January 29, 2020. The questionnaire was uploaded to the platform, which automatically generates a network link. The link was then posted via the researcher’s social media account and the organization’s website, inviting people to answer the questions and forward the questionnaire. Excluded the close contacts, a total of 16,616 questionnaires from general public were collected in this study, with 1,551 questionnaires from medical workers and 1,554 questionnaires answered in less than 200 s or by one aged less than 16 years or more than 100 years old, which were deleted. A total of 13,511 valid questionnaires were left, with a response rate of 81.3%. The samples cover 18 cities in Henan Province, China. Among the participants, there are 4,267 males (31.6%) and 9,244 females (68.4%), their average age are 32.10 ± 11.11, with the largest being 77 years old and the youngest 16 years old. Among the participants, 2,930 (21.7%) have a high school education and below, 2,761 (20.4%) have a junior college degree, and 7,820 (57.9%) have a bachelor’s degree or above. 1,900 (14.1%) have healthcare workers in their family, while 11,611 (85.9%) had none.

### Measures

The self-compiled COVID-19 Social Mentality Questionnaire was used as a measurement tool in this study (Chen et al., 2020). The questionnaire was compiled by psychological professors and doctoral students at the early stage of the COVID-19 pandemic after referring to previous researches on the Severe Acute Respiratory Syndrome (SARS) epidemic and relevant literature on sudden public health events (Qian et al., 2003; Shi et al., 2003; Shi and Hu, 2004; Xu et al., 2005; Xie et al., 2009). The questionnaire has six contents: (1) risk level during the COVID-19 pandemic, (2) social support during the COVID-19 pandemic, (3) knowledge of COVID-19, (4) self-efficacy in seeking help during the COVID-19 pandemic, and (5) the public panic during the COVID-19 pandemic. After determining the basic framework, the team members modified and improved the questionnaire through several discussions, and screened and integrated questions that were similar. Psychological scholars and postgraduates were then invited to conduct a pilot test, and the questionnaire was refined and processed according to their feedback, which was used to formulate the final questionnaire. The final questionnaire was then uploaded to the online platform wjx.cn and was tested among a large population.

#### Risk Level

Risk level is measured by a self-rated question that asks, “Have you found any cases or suspected cases of COVID-19 around you?” The answer “yes” is scored as 1, and the answer “no” is scored as 0.

#### Objective Social Support

Objective social support is measured by a self-rated question that asks, “whether someone in the family is a health care worker.” The answer “yes” is scored as 1, and the answer “no” is scored as 0.

#### Pandemic-Related Knowledge

The sub-questionnaire “Cognition Questionnaire on COVID-19 Pandemic” in the self-compiled COVID-19 Social Mentality Questionnaire was used as the measurement tool for pandemic-related knowledge. The questionnaire mainly consisted of 8 items, which respectively examined the cognition of the participants on the characteristics of COVID-19 infection, the main symptoms, the route of transmission, the knowledge of prevention and the difference between the symptoms and the common cold/flu, as well as the research progress related to the disease and the development stage of the pandemic (see Appendix 1). The score range is from 0 to 8; “very unclear” and “relatively unclear” answers are counted as 0, and “very clear” and “relatively clear” answers are counted as 1, then the total score is calculated. The Cronbach’s alpha for this questionnaire was.697.

#### Self-Efficacy

The sub-questionnaire “The Public’s Self-Efficacy in Seeking Help During the COVID-19 Pandemic” in the self-compiled COVID-19 Social Mentality Questionnaire was used as the measurement tool for self-efficacy. The public’s self-efficacy during the pandemic includes four items, which respectively examine people’s information acquisition efficacy, information identification efficacy, medical treatment acquisition efficacy and psychological assistance acquisition efficacy. The answer “yes” is counted as 1 score, “no” and “uncertain” as 0, then the total score is calculated. The Cronbach’s alpha for this questionnaire was = 0.750.

#### Public Panic

Projection measurement was used to measure public panic. The proportion of people around the participants that felt panic actually reflected the degree of panic of the subjects themselves. Participants were asked to answer the question, “How many people around you feel panic about COVID-19?” The 5 Likert scale was adopted, which indicated the participants’ panic from low to high.

### Data Analysis

SPSS 25.0 was used to analyze the collected data, and a descriptive analysis was used to describe public panic and other studied variables. Chi-square test was used to examine the existing differences in panic under different factors. Multivariate stepwise regression was conducted to explore how public panic was affected by other research variables.

## Results

### The Distribution of Panic Among the Population During COVID-19

As shown in Table 1, generally 7,291 (53.96%) people thought that more than half of the people around them experienced panic, while only 1,442 (10.67%) people thought that people around them did not. The mean of public panic was 2.99 and the standard deviation was 1.28. Chi-square test results showed that there were significant differences in the public panic mood in terms of gender (χ^2^ = 115.09, *p* < 0.001), age (χ^2^ = 515.14, *p* < 0.001), educational background (χ^2^ = 462.59, *p* < 0.001), objective social support (χ^2^ = 28.97, *p* < 0.001), risk level (χ^2^ = 59.01, *p* < 0.001), pandemic knowledge (χ^2^ = 111.46, *p* < 0.001), and self-efficacy (χ^2^ = 263.36, *p* < 0.001).

**TABLE 1:** Descriptive statistics and Chi-square test of public panic.

		Panic (*N* = 13,511, *M* = 2.99, *SD* = 1.28)					
		None (*n* = 1,442)	A small part (*n* = 4,778)	About half (*n* = 1,730)	A large part (*n* = 3,551)	Basically all (*n* = 2,010)	χ^2^
Gender	Female	867(60.1%)	3,126(65.4%)	1,209(69.9%)	2,596(73.1%)	1,446(71.9%)	115.09***
	Male	575(39.9%)	1,652(34.6%)	521(30.1%)	955(26.9%)	564(28.1%)	
Age	*M* ± *SD*	33.65 ± 11.23	32.70 ± 11.56	29.82 ± 10.73	31.60 ± 10.73	32.28 ± 10.48	515.14***
Education	*M* ± *SD*	2.11 ± 0.86	2.45 ± 0.77	2.52 ± 0.73	2.41 ± 0.81	2.13 ± 0.89	462.59***
Social support	No	1,302(90.3%)	4,094(85.7%)	1,468(84.9%)	3,010(84.8%)	1,737(86.4%)	28.97***
	Yes	140(9.7%)	684(14.3%)	262(15.1%)	541(15.2%)	273(13.6%)	
Risk level	Low	1,417(98.3%)	4,551(95.2%)	1,612(93.2%)	3,339(94.0%)	1,874(93.2%)	59.01***
	High	25(1.7%)	227(4.8%)	118(6.8%)	212(6.0%)	136(6.8%)	
Pandemic-related knowledge	*M* ± *SD*	6.54 ± 1.56	6.65 ± 1.35	6.66 ± 1.41	6.60 ± 1.36	6.55 ± 1.42	111.46***
Self-efficacy	*M* ± *SD*	3.16 ± 1.23	2.98 ± 1.28	2.79 ± 1.34	2.65 ± 1.40	2.69 ± 1.41	263.36***

*^∗∗∗^P < 0.001.*

### Correlation Analysis

The descriptive statistics and correlation matrix of each research variable are shown in Table 2. Panic is significantly and positively correlated with objective social support (*r* = 0.023, *p* < 0.01) and risk level (*r* = 0.055, *p* < 0.01), and is negatively correlated with gender (*r* = −0.086, *p* ≤ 0.01), age (*r* = 0.044, *p* < 0.01), educational background (*r* = 0.030, *p* < 0.01), and efficacy (*r* = 0.125, *p* < 0.01). There was no significant correlation between panic and pandemic knowledge.

**TABLE 2:** Descriptive statistics and correlation analysis of research variables (*N* = 13,511).

	*M* ± *SD*	1	2	3	4	5	6	7	8
1. Gender	0.32 ± 0.47	1.000							
2. Age	32.08 ± 11.09	0.039^∗∗^	1.000						
3. Education	2.36 ± 0.82	−0.049^∗∗^	−0.102^∗∗^	1.000					
4. Social support	0.14 ± 0.35	−0.009	0.019^∗∗^	0.111^∗∗^	1.000				
5. Risk level	0.05 ± 0.22	−0.010	−0.078^∗∗^	0.060^∗∗^	0.045^∗∗^	1.000			
6. Pandemic-related knowledge	6.61 ± 1.39	−0.040^∗∗^	0.035^∗∗^	0.099^∗∗^	0.043^∗∗^	−0.032^∗∗^	1.000		
7. Self-efficacy	2.85 ± 1.34	0.093^∗∗^	0.018^∗∗^	0.012	0.034^∗∗^	−0.043^∗∗^	0.298^∗∗^	1.000	
8. Panic	2.99 ± 1.28	−0.086^∗∗^	−0.044^∗∗^	−0.030^∗∗^	0.023^∗∗^	0.055^∗∗^	−0.010	−0.125^∗∗^	1.000

*^∗∗^P < 0.01.*

### Multivariate Regression Analysis

In order to further study the influencing factors of public panic under the COVID-19 pandemic, we took panic as a dependent variable and conducted multiple linear regression analysis with gender, age, education, objective social support, risk level, pandemic knowledge and self-efficacy as independent variables. The stepwise method was used to determine the main factors and the criteria was set as “Probability-of-F-to-enter ≤0.05, Probability-of-F-to-remove ≥0.10.” As shown in Table 3, among the seven models obtained by stepwise regression, the *R*^2^ (0.028) value of the 7th model was the highest, which was selected as the final model. Multivariate regression analysis results show that risk level (β = 0. 048, *p* < 0. 001), pandemic knowledge (β = 0. 030, *p* < 0.01), and objective social support (β = 0. 029, *p* < 0. 01) can positively predict panic, while self-efficacy (β = −0. 125, *p* < 0. 001), gender (β = −0. 073, *p* < 0. 001), and education (β = −0. 045, *p* < 0. 001), age (β = −0. 041, *p* < 0. 001) negatively forecast panic.

**TABLE 3:** Multivariate regression analysis of the influencing factors of public panic.

	Model 1		Model 2		Model 3		Model 4		Model 5		Model 6		Model 7	
	β (95%CI)	*t*	β (95%CI)	*t*	β (95%CI)	*t*	β (95%CI)	*t*	β (95%CI)	*t*	β (95%CI)	*t*	β (95%CI)	*t*
Self-efficacy	−0.125 (−0.135, −0.103)	−14.616^∗∗∗^	−0.118 (−0.128, −0.096)	−13.777^∗∗∗^	−0.116 (−0.126, −0.094)	−13.541^∗∗∗^	−0.115 (−0.125, −0.094)	−13.465^∗∗∗^	−0.115 (−0.125, −0.093)	−13.415^∗∗∗^	−0.124 (−0.135, −0.101)	−13.836^∗∗∗^	−0.125 (−0.135, −0.102)	−13.922^∗∗∗^
Gender			−0.075 (−0.252, −0.160)	−8.758^∗∗∗^	−0.075 (−0.252, −0.159)	−8.735^∗∗∗^	−0.076 (−0.256, −0.164)	−8.939^∗∗∗^	−0.075 (−0.253, −0.161)	−8.793^∗∗∗^	−0.073 (−0.247, −0.155)	−8.538^∗∗∗^	−0.073 (−0.247, −0.155)	−8.523^∗∗∗^
Risk level					0.049 (0.184, 0.375)	5.753^∗∗∗^	0.051 (0.196, 0.387)	5.997^∗∗∗^	0.048 (0.180, 0.371)	5.66^∗∗∗^	0.049 (0.184, 0.375)	5.742^∗∗∗^	0.048 (0.177, 0.368)	5.597^∗∗∗^
Education							−0.035 (−0.082, −0.029)	−4.164^∗∗∗^	−0.039 (−0.088, −0.035)	−4.589^∗∗∗^	−0.042 (−0.093, −0.040)	−4.919^∗∗∗^	−0.045 (−0.098, −0.045)	−5.252^∗∗∗^
Age									−0.039 (−0.006, −0.003)	−4.567^∗∗∗^	−0.040 (−0.007, −0.003)	−4.711^∗∗∗^	−0.041 (−0.007, −0.003)	−4.817^∗∗∗^
Pandemic-related knowledge											0.031 (0.012, 0.045)	3.453^∗∗^	0.030 (0.012, 0.044)	3.376^∗∗^
Social support													0.029 (0.043, 0.167)	3.334^∗∗^
*R*^2^	0.016		0.021		0.024		0.025		0.026		0.027		0.028	
Δ*R*^2^	0.016		0.006		0.002		0.001		0.002		0.001		0.001	
*F* change	213.640^∗∗∗^		76.708^∗∗∗^		33.091^∗∗∗^		17.34^∗∗∗^		20.859^∗∗∗^		11.923^∗∗^		11.116^∗∗^	

*^∗∗^P < 0.01, ^∗∗∗^P < 0.001, Dependent Variable: Panic.*

### Markov Chain Monte Carlo (MCMC) Bayesian Estimation

Compared with the maximum likelihood estimation, the Bayesian estimation could benefit to evaluate the complicated likelihood functions and posteriors in model estimation. According to Washington et al. (2011), when the posterior estimates in a model are resulted from the statistical likelihood and the prior with a random sample, the Bayes’ theorem would be adopted to estimate the model. Therefore, a standard Markov Chain Monte Carlo (MCMC) sampling method is needed to simulate the posterior densities of the model under the Bayesian framework. However, as the model was intractable in analysis, following the suggestions of Bolduc et al. (2005), a MCMC Bayesian estimation was applied to conduct the posterior inferences of the results from stepwise regression in this paper. As shown in Table 4, the results of stepwise regression were consistent with the positive and negative coefficients of explanatory variables in the results of MCMC method, and the difference was not significant. In stepwise regression, coefficients of explanatory variables other than age were all within 95% Credible Interval of MCMC method.

**TABLE 4:** Results of Markov Chain Monte Carlo (MCMC) Bayesian estimation.

	Mean	*SD*	95% credible interval
Self-efficacy	−0.12	0.01	(0.14, −0.10)
Gender	−0.05	0.01	(−0.06, −0.04)
Risk level	0.07	0.01	(0.04, 0.09)
Education	−0.04	0.01	(−0.05, −0.02)
Age	0.00	0.00	(0.00, 0.00)
Pandemic related knowledge	0.06	0.02	(0.02, 0.09)
Objective social support	0.03	0.01	(0.01, 0.04)

*Dependent Variable: Panic.*

## Discussion

Public panic is an objective response to major risk events occurring on the public, but it is negative in nature and often causes more harm than good. If the spread of public panic cannot be controlled in a timely manner, it may cause a negative chain reaction, which will not only adversely affect the stability and management of the whole society, but also hinder the prevention and control process of the pandemic. Therefore, analyzing the influencing factors of public panic under the pandemic situation could help people find ways to overcome panic and help administration departments make scientific decisions and carry out pandemic prevention and control more effectively.

### Overview (Profile) of the Public Panic

The COVID-19 outbreak has had a huge psychological impact on the population. From the psychological projection perspective (Cai and Shen, 2010), sometimes people unconsciously reflect their emotions, attitudes and thoughts toward external things or others. Therefore, the perceived panic around a certain subject may reflect a certain degree of unawareness of one’s own feelings of panic. The results of this study showed that 89.33% of the public believed that someone around them experienced panic, and the average measurement of public panic was 2.99 ± 1.28 (*M* ± *SD*), indicating a high level of panic. Due to the lack of clear understanding and control over COVID-19, the theory of psychological stress explains that COVID-19 is a relatively serious stress event for both social groups and individuals. The conclusions are consistent with the relevant studies during the SARS period. Shi and Hu (2004) found through investigation that in the early stage of SARS, people often made irrational evaluations on the current situation and consequences of the epidemic, which led to panic. Xie et al. (2005) indicated that the level of psychological anxiety of the public generally increased during the SARS epidemic, and majority of the public’s psychological anxiety turned into psychological panic due to the failure to receive effective feedback. It can be seen that panic is a normal and objective response to a major epidemic, yet, this seemingly completely negative emotional response actually has some positive meaning. Studies have shown that in life-threatening situations, negative emotions narrow the range of individual’s momentary thought-action repertoire, improve people’s ability to act quickly and firmly, and thus increase the survival probability of individuals, which has evolutionary significance (Xie, 2019).

Therefore, the purpose of dealing with the negative emotional reaction such as panic should not be aimed at total elimination, but to carry out reasonable guidance for such emotion, so that people can not only maintain reverence for nature, but also protect their mental health. Here, it is suggested that the administrative departments must actively encourage scientific research on psychological changes of the public during the pandemic, and formulate reasonable policies to effectively channel the public’s panic, maintain social stability and speed up the process of fighting against the pandemic.

### The Role of Self-Efficacy

The results show that self-efficacy can negatively predict panic, that is, the higher an individual’s level of self-efficacy, the less likely they are panic. According to the social cognitive theory, self-efficacy is the degree to which an individual is confident in his or her ability to complete a task or behavior; whether a person can engage in a certain activity smoothly and successfully is affected by his self-efficacy because an individual’s feeling of self-efficacy restricts their motivation level, behavior mode of activities and various psychological levels (Bandura et al., 1999). The self-efficacy investigated in this study is described as one where, during the COVID-19 pandemic, an individual initiates a request for help, and during the request process, the prediction of the outcome of the request reflects the individual’s confidence level to complete it. In the process of seeking help, individuals with high self-efficacy can further improve their problem-solving ability and have more confidence when facing tasks and difficulties in the future (Williams and Takaku, 2011). At the same time, individuals with high self-efficacy will perceive more positive effects of help-seeking behavior, so their psychological cost in the process of help-seeking is also relatively low (Nadler, 1991). They are more likely to seek help to relieve stress (Aspinwall and Taylor, 1992), which helps reduce their anxiety, depression, panic and other emotional problems caused by situational factors, and maintain their emotional stability.

In this regard, it is recommended that the government should further broaden the channels of medical and psychological assistance for the public, encourage more social forces to invest in the fight against COVID-19, and transmit positive social energy while employing correct adverse actions in the fight against COVID-19 to show determination to overcome the pandemic, thereby boosting public confidence to increase the effectiveness of the fight against the pandemic.

### The Role of Gender

Gender is an effective indicator of panic; women are more likely to panic in the face of a pandemic. It may be related to the personality traits of women, such as sensitivity (Su and Wang, 2014). Therefore, when experiencing changes in their social environment, women are more likely to perceive the emotional state and changes of the people around them, making them feel more panic. It may also be related to the cognitive characteristics of females. Studies have shown that females are dominant in the emotional and intuitive dimensions, while males are dominant in the ideological and sensory dimensions (Peng et al., 2006). Therefore, women’s emotions are more likely to be affected by stressful events. During the SARS epidemic, some researchers found that the degree of panic, stress intensity and stress influence of female college students were significantly higher than male college students (Yin et al., 2003). In addition, according to the emotional contagion theory (Doherty, 1997), women have higher emotional sensitivity than men; therefore, in a pandemic, women are more susceptible to being emotionally affected by the people around them, leading to panic and other negative emotions. It is consistent with the results of this study, which showed that the proportion of female college students who perceived panic around them is significantly higher than that of male college students (Zhao et al., 2020).

It is suggested to pay attention to the differences between different gender groups, fully understand their psychological needs, and carry out targeted psychological assistance.

### The Role of Risk Level

Risk level can also effectively predict panic, that is, the higher the risk level, the greater the possibility of panic. According to the mental model of risk (Svenson, 1988), when the risk faced by an individual exceeds the level of acceptance, a strong physical and mental reaction may occur, such as panic, anxiety and other adverse psychological reactions. The degree of risk perceived by an individual is negatively correlated with its geographic location; the farther the location of the pandemic, the lower the degree of risk. Because the geographical location of COVID-19 is close to people, it is also a reflection of psychological distance. Therefore, when confirmed/suspected cases appear around individuals, people will feel more danger and threat, and the emotional reaction of panic and anxiety will be stronger (Qian et al., 2003; Xie et al., 2009). Previous research has also confirmed this conclusion; Qian et al. (2003) found that during the SARS epidemic, when SARS first appeared in their city, people’s negative emotions increased significantly, and when SARS appeared in the school, company or community, the spatial and psychological distance between SARS and the people was further reduced, and people showed more tension, fear, pessimism and helplessness.

The current situation of the COVID-19 pandemic worldwide is still very serious, thus, the government should adopt active prevention and control policies and concentrate its efforts on high-quality allocation of resources on the basis of science. Meanwhile, all sectors of society should work together, help each other, exchange needed goods and make joint efforts to reduce the risk level.

### The Role of Education and Age

Education background and age can also negatively predict panic, that is, the more highly educated people are, the less likely they panic, and the older the people are, the less they panic. In this study, samples were taken in the early stage of the pandemic outbreak, which was also a time when information was expanding rapidly and everyone was in an information storm. According to the signal theory (Spence, 2002), a large amount of information flooding in a short time produces information explosion. Information noise brought by a large amount of uncertainty and information redundancy causes information damage, thus disturbing people’s ability to differentiate information (Miao and Zhu, 2006). In addition, the pandemic is an extraordinary period, and this special situational factor naturally becomes a condition for the formation and living space of rumors. Highly educated participants tend to have higher cognitive level, more information acquisition channels and stronger information collection ability (Xu et al., 2005), which helps them identify misinformation more effectively, obtain practical and effective coping strategies, avoid being misled by rumors and reduce unnecessary panic. Moreover, the aging rate of memory of highly educated people is low (Feng, 2005). The memory of overcoming SARS also brings them more positive mental motivation, which buffers their feelings of panic about COVID-19.

In terms of age, the research results show that age and educational background are significantly positively correlated, that is, the older the subjects are, the higher their educational background is. Combined with the previous discussion, it explains why the older the participants are less likely to panic. In addition, according to the social learning theory, individual concepts of reality are derived from the process of comparative verification of these concepts against some other real criteria (Bandura, 1986). In the same fuzzy information condition, for individuals with direct experience, although the direct experience itself is not pleasant or even compulsive, the direct experience will provide the individual with corresponding objective feelings, which can often correct the unnecessary psychological panic caused by information ambiguity (Xie et al., 2005). In this study, the older subjects may have experienced SARS, H1N1 and other infectious diseases, and the accumulated knowledge and experience makes them more informed and confident in coping with COVID-19, resulting in less panic.

### The Role of Pandemic-Related Knowledge and Objective Social Support

Surprisingly, pandemic-related knowledge and objective social support can positively predict panic, that is, people with more pandemic-related knowledge and objective social support are more likely to panic. However, reviewing the literature, it is found that the results are consistent with previous studies.

First of all, in terms of pandemic-related knowledge, the sampling time of this study was in the early outbreak of the pandemic, and it could not be ruled out that some participants misjudged rumors as knowledge, thus their panic level was relatively high. Shi and Hu (2004) pointed out in their study that the relevant information in the early stage of the pandemic had a strong negative effect on the public, which would significantly increase the public’s perception of risk. Moreover, according to the social learning theory, the individual’s conception of reality is derived from the process of comparative verification of these concepts and some other real standards (Bandura, 1986). Therefore, personal experience is of great significance to risk cognition, and subjects without direct personal experience are more likely to be induced by external information and thus produce corresponding psychological responses (Wiegman et al., 1991). Qian et al. (2003) conducted a survey of the Beijing population during the SARS epidemic and found that in the first 2 weeks of the outbreak, due to the explosion of all kinds of information and people’s lack of knowledge about SARS and the epidemic, people could not effectively distinguish facts and rumors and were inevitably misled. Therefore, the more information they received, the more panic they felt. In addition, Xie et al. (2005) pointed out that for individuals without direct experience, when the information provided by the outside world is not of clear guiding significance, individuals are likely to have adverse psychological reactions such as anxiety and panic. Moreover, at the outbreak period of the pandemic, information closely related to the public itself and negative information were of utmost concern to the public. At the beginning of a crisis event, the public tends to make a judgment on the risk of the event based on objective indicators such as the frequency of the occurrence and the severity of the consequences, which naturally leads to negative emotions such as panic (Shi et al., 2003).

In terms of objective social support, an operational definition of objective social support in this study is, “Is there anyone in the family engaged in medical care?” In the background of a major pandemic, the medical staff are fighting in the front line of resistance to the disease, and especially under the highly contagious COVID-19, the concern of family members for the occupational health of medical staff may result to a certain level of anxiety. Moreover, according to the spillover theory (Staines, 1980), medical workers are likely to share what they have seen and heard at the front line with their families; combined with the uncertainty of various information during the pandemic period, it is very likely that their families will experience a strong sense of panic.

Thus, it is recommended to adopt a diversified approach to spread accurate and timely information about the COVID-19 pandemic, repel rumors with factual reporting, and reduce panic among the public. Besides, in order to reduce the impairment of rumors and misinformation, it is highly necessary to call for psychological research to reveal the public’s cognition towards the COVID-19 pandemic in time (Castelnuovo et al., 2020).

## Clinical Implications

Combined with the results, here are proposed three clinical recommendations for the general public during the COVID-19 pandemic.

First, it is a normal emotional response to feel panic when facing with great dangers, such as during COVID-19, and such response can help people staying away from dangers. When panic occurs, accept the existence of the emotion is initial and essential, and then make every effort to seek and practice the scientific solution, that is, adopt the standard science-based self-protection implements. For example, stay at home and do not to going out, gathering and visiting others. Besides, it is an obligation to wear a medical surgical/protective facial mask if someone has to go outside. Further, washing hands in accordance with the Seven-step method frequently, keeping the room ventilated, and eat fully cooked food during the COVID-19 pandemic are crucial personal protections. The proper personal protection can greatly reduce the risk of being infected.

Second, in the early stage of pandemic, people often panic because of excessive attention to the pandemic and ineffective screening of misinformation. Here, it is suggested to acquiring the pandemic-related knowledge/information through the authoritative media. A clear and objective understanding of the development of the pandemic can help people to reduce panic. Meanwhile, in times of emergency, the greatest contribution to society is to manage oneself well without spreading or believing rumors.

In addition, studies have shown that only the pandemic-related knowledge acquisition is insufficient in reducing panic, rather than that, making the public to be more aware of the prevention and control measures seems to be more important to confine the pandemic (Liu et al., 2004), as it can effectively improve people’s self-efficacy, thereby reducing the damage of COVID-19 pandemic to the people’s mental health (Wang et al., 2020). At present, scientific analysis and interpretation of COVID-19 related information, guidance of public opinion correctly, clear prevention methods are of great importance to improving the psychological state of the public. In the long term, it is strongly recommended to improve people’s public health literacy (Huang et al., 2015).

## Limitation

Due to the limitations of the overall sample size and the actual situation, this study adopts the on-line convenient sampling method. Therefore, due to the sample distribution, the representativeness is limited. The number of previous studies available for reference was limited owing to the unexpected and unknown nature of the COVID-19 pandemic; moreover, as time was limited, the self-compiled questionnaire used in this study was relatively crude. In addition, this study adopts a cross-sectional study design, which cannot fully reflect the psychological development of the public during the pandemic. It is suggested that future studies should adopt a longitudinal study design or mixed study design, in order to conduct a comprehensive and in-depth study of the psychological development of the public in emergencies.

## Data Availability Statement

The raw data supporting the conclusions of this article will be made available by the authors, without undue reservation.

## Ethics Statement

The studies involving human participants were reviewed and approved by the Henan University Institutional Review Board. Written informed consent to participate in this study was provided by the participants’ legal guardian/next of kin.

## Author Contributions

YL and SW were the principal investigators for the study, generated the idea and designed the study. XN and KF were the primary writers of the manuscript, and approved all changes. KF and SW supported the data input and data analysis. YL and XN supported the data collection. All authors were involved in developing, editing, reviewing, and providing feedback for this manuscript and have given approval of the final version to be published.

## Conflict of Interest

The authors declare that the research was conducted in the absence of any commercial or financial relationships that could be construed as a potential conflict of interest.

## Appendix 1

The 15 items involved in this study are listed below:

Objective social support: “Is someone in your family a healthcare worker?”

Public panic: “How many people around you feel panic about COVID-19?”

Risk level: “Are there confirmed or suspected cases in your area?”

Pandemic-related knowledge:

a) Do you know the main symptoms of COVID-19?
b) Do you know how COVID-19 is transmitted?
c) Do you know the difference in symptoms between COVID-19 and the common cold?
d) Are you aware of the current pandemic?
e) Are you aware of current research progress on COVID-19?
f) Do you think wearing a mask can prevent COVID-19 infection?
g) Do you know how to wash your hands properly?
h) Do you think that the behaviors of dining and gathering is at risk of COVID-19 infection?

Self-efficacy:

a) I am sure I have the resources I can use to gain knowledge about COVID-19.
b) I’m sure I know how to distinguish the rumor from the truth.
c) I’m sure I know how to get proper medical treatment if I need it.
d) I’m sure I know how to get the proper psychological services if I need it.
